# Community health workers improve contact tracing among immigrants with tuberculosis in Barcelona

**DOI:** 10.1186/1471-2458-12-158

**Published:** 2012-03-06

**Authors:** Jesús Edison Ospina, Àngels Orcau, Juan-Pablo Millet, Francesca Sánchez, Martí Casals, Joan A Caylà

**Affiliations:** 1 Epidemiology Service, Public Health Agency of Barcelona, Plaza Lesseps 1, 08023 Barcelona, Spain; 2 Departament de Pediatria, Ginecologia i Medicina Preventiva, Universitat Autònoma de Barcelona (UAB), Plaza Cívica-Campus de la UAB 08193 Bellaterra, Spain; 3 CIBER Epidemiología y Salud Pública (CIBERESP), C/Melchor Fernández Almagro 3-5, 28029, Madrid, Spain; 4 Servicios de Medicina Interna, Enfermedades Infecciosas y Microbiología del Hospital del Mar Barcelona, Paseo Marítimo 25-29, 08003 Barcelona, Spain; 5 Departament de Salut Pública, Universitat de Barcelona, Gran Via de les Corts Catalanes, 585, 08007 Barcelona, Spain; 6 Departament de Ciencies Basiques, Universitat Internacional de Catalunya, Josep Trueta s/n 08195 Sant Cugat del Vallés, Spain

## Abstract

**Background:**

The important increase in immigration during recent years has changed the epidemiology and control strategies for tuberculosis (TB) in many places. This study evaluates the effectiveness of intervention with community health workers (CHW) to improve contact tracing among immigrants.

**Methods:**

The study included all TB cases detected by the Barcelona TB Program from 2000 to 2005 and compared a period without CHW intervention (2000-2002) to a period with CHW intervention (2003-2005). The influence on contact tracing of sex, age, hospital of diagnosis, district of residence, birthplace, HIV, homeless and CHW intervention was analysed by logistic regression. *Odds ratio *(OR) and 95% confidence intervals (CI) were calculated.

**Results:**

960 foreign born TB cases were detected, 388 in the intervention period. Contact tracing was performed on 65,7% of 201 smear-positive cases during the pre-intervention period compared to 81.6% of 152 smear-positive TB cases during the intervention period (*p *< 0.001). Risk factors associated with incomplete contact tracing of smear-positive index cases included being diagnosed in two hospitals without contact tracing TB unit (OR = 3.5; CI:1.4-8.9) and (OR = 4.6; CI:1.6-13.5) respectively, birth place in India-Pakistan (OR = 4.4; CI:1.9-10.3) or North Africa (OR = 4.3; CI:1.8-10.5), having an unknown residence (OR = 5.4; CI:1.6-18.0), being HIV-infected (OR = 6.1; CI:2.5-14.8) or homeless (OR = 3.3; CI:1.3-8.2), and the absence of CHW intervention (OR = 2.4; CI:1.3-4.3).

**Conclusions:**

The effectiveness of contact tracing for TB control in areas with high immigration can be improved by incorporating CHWs who act as translators, cultural mediators and facilitators who accompany cases and contacts through treatment and follow-up.

## Background

Tuberculosis (TB) is one of the most important causes of infectious disease mortality worldwide, particularly in low income countries. The incidence of TB has stabilised or declined in most of the regions defined by the World Health Organization (WHO), but the total number of new cases continues to rise slowly due to population growth [1]. According to the WHO in 2010, there were an estimated 8.8 million incident cases of TB (range, 8.5 million-9.2 million) globally, equivalent to 128 cases per 100 000 population. Most of the estimated number of cases in 2010 occurred in Asia (59%) and Africa (26%); smaller proportions of cases occurred in the Eastern Mediterranean Region (7%), the European Region (5%) and the Region of the Americas (3%). The five countries with the largest number of incident cases in 2010 were India (2.0 million-2.5 million), China (0.9 million-1.2 million), South Africa (0.40 million-0.59 million), Indonesia (0.37 million-0.54 million) and Pakistan (0.33 million-0.48 million). India alone accounted for an estimated one quarter (26%) of all TB cases worldwide, and China and India combined accounted for 38%. In addition, 1.1 million (range, 0.9-1.2 million) deaths from TB among HIV-negative people and an additional 0.35 million (range, 0.32-0.39 million) deaths from HIV-associated TB [2]. Globally, it is estimated that 3.3% of all new TB cases had MDR-TB in 2009 and each year an estimated 440,000 new MDR-TB cases emerge and 150,000 persons with MDR-TB die [3].

In many European countries, immigration, particularly from high TB burden countries, has increased. In January 2010, 5.7 million of foreign-born persons were registered in Spain (12.2% of the total population), in 1999 were registered 748.953 (1.8% of the total population), this representing an increase of over three million people in eleven years [4]. These percentages have been even higher in large cities such as Barcelona or Madrid, where the immigrant population has reached 17.6% and 17.1%, respectively [5,6].

This demographic change has had an important impact on TB in Barcelona, provoking a slower decline in TB incidence [7]. The strategy adopted by the Barcelona TB Control Program (TBPCP) in 1987, with public health nurses (PHN) to follow the patients and coordinate contact tracing achieved indicators of good control in later years. Until 2003 treatment completion of the native patients was over 85% and contact tracing among smear positive patients was over 88%. However, for the immigrant population the contact tracing was under 50% during these years [8].

To improve the follow-up of the immigrant TB patients and their contacts, according to international guidelines [9] in January 2003 the TBPCP began an intervention strategy using community health workers (CHW). They work in coordination with PHN and health-care personnel in activities targeted to improve treatment adherence, contact tracing, outbreaks control in domestic, occupational and leisure settings [10]. The aim of the present study is to assess the effectiveness of the CHW strategy in improving the contact tracing by comparing a period with CHW intervention to one without in a city with massive recent immigration.

## Methods

### Study design

Quasi-experimental study historical (pre-post) comparing the pre-intervention period from 2000-2002 (with only PHN) with the intervention period, 2003-2005 (CHW and PHN intervention). Contact tracing was compared between both periods.

### Study population

All TB cases registered by the TBPCP between January 1^st ^2000 and December 31^st ^2005, residents in the city of Barcelona were included.

### Variables

The study of associated factors involved in performing contact tracing included socio-demographic characteristics (sex, age, hospital of diagnosis: all hospitals had diagnostic services and performed patient monitoring, but hospitals B and D had no contact tracing team and these were refered to their respective general practitioner (GP); geographical area of origin and district of residence), risk factors (injecting drug use, HIV infection, smoking: consumption of one or more cigarettes per day; use of alcohol: consumption of over 280 g of alcohol per week for men and over 168 g for women; incarceration history, homeless), clinical data (type of TB and radiological results) and use of CHW intervention.

### Case definition

A case was defined as an individual who is diagnosed with TB disease and is prescribed anti-TB treatment, including those who prematurely discontinue treatment for any reason [11].

### Contact tracing performed

When a new TB case is detected in a health-care centre, the information is sent to the TBPCP of Barcelona. The healthcare team evaluates the need for CHW intervention depending on the specific problems presented by patients and each case is assigned to a PHN and to a CHW, depending on their birthplace, language, culture and any other needs of cases and their contacts.

Contact tracing was defined as performed when at least one contact was traced for each TB patient. The smear-positive pulmonary TB were prioritised [12,13]. Given the low coverage of contact tracing performed observed in immigrant population in the pre-intervention period, our objective was to get the 70% of coverage in the intervention period with all cases that involved the CHW.

### Community health workers

CHW are professionals who are members of their target population and also integrated within the healthcare team. They come from the communities themselves and have been specifically trained in TB and psycho-social skills, with the purpose of connecting immigrant patients to the healthcare system. Five CHW were selected from each immigrant community and worked a specific number of hours per week according to the number of cases: Asia (Pakistan, India, Bangladesh), 12 hours (112 cases); North Africa (Morocco, Algeria, Tunesia and arab countries), 20 hours (70 cases); Sub-Saharan Africa, 12 hours (32 cases); China, 6 hours (22 cases) and Latin America, 20 hours (152 cases). They were also involved with reported cases from other countries. For the distribution of the working hours of the CHW it was taken into account that the CHW from Latin America and Asia (Pakistan, India, Bangladesh), were better trained given their previous experience in other programmes and that the patients of these countries, were more concentrated in certain districts and neighborhoods of the city, which made the approach and contact tracing easies. The CHW from North Africa intervened with many other patients from other countries, since he was able to speak Arabic, French and English. In addition the previous control of other diseases in individuals from these countries, showed that there were major difficulties for treatment compliance, medical control and location at patients homes. Also many cases were IDUs.

The mechanisms used to validate the CHW information contact tracing performed were: weekly meetings with the team of public health nursing, ongoing monitoring of program coordinator with each CHW and monthly meetings with experts from DOTs. CHW activities fall in three fundamental areas, always in collaboration with PHN [14]:

1. **Support of healthcare teams: **Active follow-up of cases and contacts, with visits to the cases houses, accompanying patients to appointments, providing counseling and information on treatments.

2. **Health information: **Educational sessions in healthcare centres, private homes and immigrant associations, using a 30-minute video about TB in Arabic, Spanish and Urdu and brochures on the disease translated into seven languages [15].

3. **Community mobilisation: **Assistance for obtaining a residence permits, housing, food banks, public dining halls and a health-card application.

### Statistical analysis

A descriptive analysis was performed by calculating proportions. The median and interquartile range were calculated for quantitative variables. Results for each group were analysed in terms of if contact tracing was performed or not. Categorical variables were compared using the χ **2 **test. *Odds ratios *(OR) and confidence intervals to 95% (CI) were calculated as a measure of association. The variables of epidemiological interest and those found to be statistically significant in the bivariate analysis were included. A *p*-value of < 0.05 was considered statistically significant. For multivariate analysis, a statistical logistic regression with stepwise method of variables selection was used to determine the factors associated with contact tracing. Analyses were conducted with the statistical packages SPSS, v. 13.0 (SPSS Inc., Chicago, IL, USA) and the statistical package R (The R Foundation for Statistical Computing) version 2.6.0. [16].

## Results

A total of 572 TB cases among foreign people were detected in the pre-intervention period and 388 in the intervention period. During the intervention period, 152 (39.2%) were from Latin American countries, 112 (28.9%) from India or Pakistan, 42 (10.8%) from North Africa, 16 (4.1%) from Sub-Saharan Africa, and 66 cases (17%) from other countries. CHW worked with 79.4% of these cases, 12.4% were resolved directly by the PHN and the remaining 8.2% could not be contacted. The majority of the TB cases attended by the CHW were men, between 25 and 39 years of age. Almost half lived in an inner city, socioeconomically deprived district. Pulmonary TB was the most frequent presentation (73.2%) and 39.2% cases were smear-positive cases.

**Figure 1:**
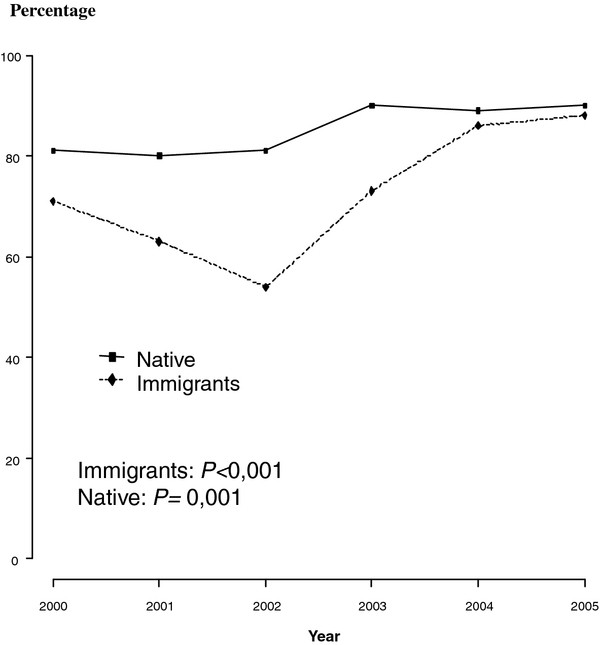
**Percentage of cases with smear-positive pulmonary tuberculosis with conventional contact tracing completed**. Barcelona 2000-2005.

**Figure 2:**
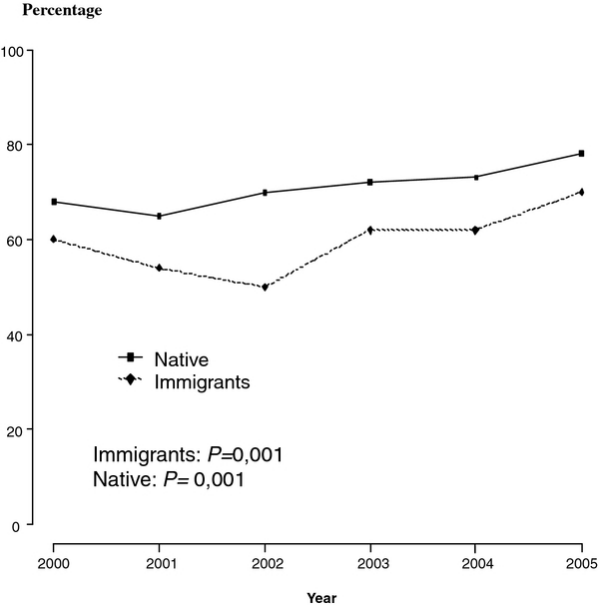
**Percentage of cases of all clinical forms of tuberculosis with conventional contact tracing completed**. Barcelona 2000-2005.

In comparing cases with CHW intervention to cases without, the CHW group had a higher rate of inner city residents, age between 25 and 39 years and a lower proportion were from North African countries. The increase in contact tracing coverage from the smear-positive pulmonary TB and all clinical forms of TB in the intervention period was statistically significant (Table 1 and Figures 1 and 2)

**Table 1:** Socio-demographic and clinical characteristics of immigrants with tuberculosis, without and with community health worker intervention.

Variables	Before CHW^a ^Intervention 2000-2002 (572 cases) N (%)	After the introduction of CHW intervention 2003-2005 (388 cases) N (%)	*p-value*
**Sex**			
Male	392 (68.5)	259 (66.8)	
Female	180 (31.5)	129 (33.2)	0.6
**Age **(median: 38; IQR^b^: 28-56)			
0**-**14	34 (6.0)	17 (4.4)	
15**-**24	112 (19.6)	58(14.9)	
25**-**39	300 (52.5)	241 (62.1)	
40 or over	125 (21.9)	72 (18.5)	< 0.001
**Geographical area of origin**			
Latin America	202 (35.3)	152 (39.2)	
India-Pakistan	136 (23.8)	112 (28.9)	
North Africa	92 (16.1)	42 (10.8)	
Other countries	142 (24.8)	82 (21.1)	< 0.001
**District of residence**			
Ciutat Vella	197 (34.4)	161 (41.5)	
Other	339 (59.3)	213 (54.9)	
Unknown	36 (6.3)	14 (3.6)	0.02
**Homeless**			
Yes	48 (8.4)	29 (7.5)	
No	524 (91.6)	359 (92.5)	0.7
**Smoking**			
Yes	183 (32.0)	116 (29.9)	
No	389 (68)	272 (70.1)	0.5
**Alcoholic**			
Yes	79 (13.8)	56 (14.4)	
No	493 (86.2)	332 (85.6)	0.2
**HIV**			
Yes	49 (8.6)	36 (9.3)	
No	523 (91.4)	352 (90.7)	0.8
**IDU^c^**			
Yes	22(3.8)	20 (5.2)	
No	550 (96.2)	368 (94.8)	0.4
**Type of TB^d ^of index case**			
Pulmonary smear-positive	201 (35.2)	152 (39.2)	
Pulmonary Smear (-) Culture (+)	115 (20.1)	84 (21.6)	
Pulmonary culture negative	96 (16.8)	48 (12.4)	
Extrapulmonary	159 (27.8)	104 (26.8)	0.22
**Chest X-ray in Pulmonary TB**			
Normal	88 (15.4)	67 (17.3)	
Cavitary	138 (24.1)	101(26)	
Non Cavitary	337 (58.9)	218 (56.2)	0.49
**CT^e^**			
Performed	317 (55.4)	257 (66.2)	
Not performed	255 (44.6)	131 (33.8)	< 0.001

Barcelona 2000-2002 and 2003-2005

^a ^Community health worker. ^b^Interquartile range. ^c^Injecting drug use. ^d^Tuberculosis. ^e^Conventional contact tracing

The most significant activities of CHW include the active-follow up in 194 TB cases and contact census, a total of 293 counseling sessions, 147 linguistic mediation session, 264 individualised and 97 group educational sessions about TB, 280 home visits, 70 hospital visits and 5,935 telephone calls (a median of 15.3 calls per case) were performed.

Factors associated with failure to conduct contact tracing for smear-positive cases include to be diagnosed in hospitals B and D (hospitals without any specifics screening services contacts), birthplace in India, Pakistan or North Africa, unknown district of residence, HIV infection, homeless and those without CHW intervention (Table 2). Factors associated with failure to conduct contact tracing for all forms of TB include male, hospitals B and D, birthplace other than Latin American countries, unknown district of residence, incarceration history, homeless, index who had culture-negative or extra-pulmonary TB or had a normal chest X-ray and no CHW intervention (Table 3).

**Table 2:** Multivariate analysis of pre-post community health workers intervention and of others factors predicting the failure to perform contact tracing among smear-positive pulmonary tuberculosis immigrant patients.

Variables	CT^a ^not performed N/Total(%) N = 353	CT OR^b ^95%CI	CT OR^c ^95%CI	CT *p*-value
**CHW intervention^d^**				
Yes (2003-2005 period)	28/152(19.0)	1	1	
No (2000-2002 period)	69/201(34.3)	2.3(1.4-3.8)	2.4(1.3-4.3)	0.005
**Sex**				
Female	18/126(14.3)	1		
Male	79/227(34.8)	3.2(1.8-1.7)		
**Age**				
0-24	17/77(22.0)	1		
25-39	56/201(27.9)	1.4(0.7-2.5)		
40-64	20/63(31.7)	1.6(0.8-3.5)		
65 years and over	4/12(33.3)	1.8(0.5-6.6)		
**Hospital of diagnosis**				
Other hospitals	12/55(21.8)	1	1	
Hospital A	16/60(26.7)	1.3(0.6-3.1)	1.1(0.4-3.0)	0.871
Hospital B	33/80(41.2)	2.5(1.1-5.5)	3.5(1.4-8.9)	0.008
Hospital C	4/52(7.7)	0.3(0.1-1)	0.5(0.1-1.9)	0.319
Hospital D	15/40(37.5)	2.1(0.9-5.3)	4.6(1.6-13.5)	0.006
Hospital E	17/66(25.8)	1.2(0.5-2.9)	0.9(0.3-2.4)	0.768
**Geographical area of origin**				
Latin America	29/167(17.4)	1	1	
India-Pakistan	18/47(38.3)	2.9(1.5-6)	4.4(1.9-10.3)	0.001
North Africa	25/49(51.0)	4.9(2.5-10)	4.3(1.8-10.5)	0.001
Other countries	25/90(27.8)	1.8(1.0-3.3)	1.4(0.7-3.0)	0.371
**IDU^e^**				
No	84/331(25.4)	1		
Yes	13/22(59.1)	4.3(1.8-10.3)		
**District of residence**				
Other districts	49/237(20.7)	1	1	
Inner city	33/95(34.7)	2.0(1.2-3.4)	1.1(0.5-2.3)	0.747
Unknown	15/21(71.4)	9.6(3.5-26)	5.4(1.6-18.0)	0.006
**HIV infection**				
No	77/321(24)	1	1	
Yes	20/32(62.5)	5.3(2.5-11.3)	6.1(2.5-14.8)	< 0.001
**Smoking**				
No	42/212(19.8)	1		
Yes	55/141(39)	2.6(1.6-4.2)		
**Use of alcohol**				
No	63/286(22)	1		
Yes	34/67(50.7)	3.65(2.1-6.4)		
**Incarceration history**				
No	87/336(25.9)	1		
Yes	10/17(58.8)	4.1(1.5-11.1)		
**Homeless**				
No	76/320(23.7)	1	1	
Yes	21/33(63.6)	5.6(2.7-12)	3.3(1.3-8.2)	0.011

Barcelona 2000-2005

^a^Contact tracing, ^b^Odds ratio, ^c ^Adjusted odds ratio, ^d^After the introduction of community health workers intervention, ^e^injecting drug user

**Table 3:** Multivariate analysis of pre-post community health workers intervention and of others factors predicting the failure to perform contact tracing among immigrants in all forms of tuberculosis.

Variables	CT^a ^Not performed N/Total(%) N = 960	CT *OR*^b ^95%CI	CT *OR*^c ^95%CI	CT *p*-value
**CHW intervention^d^**				
Yes (2003-2005 period)	131/388(33.8)	1	1	
No (2000-2002 period)	255/572(44.6)	1.6(1.2-2.0)	1.8(1.3-2.5)	< 0.001
**Sex**				
Female	72/309(23.3)	1	1	
Male	314/651(48.2)	3.0(2.3-4.2)	1.9(1.3-2.7)	0.001
**Age**				
1-14	16/51(31.4)	1		
15-24	58/170(34.1)	1.13(0.6-2.2)		
25-39	227/541(42.0)	1.6(0.9-3)		
40-64	73/170(42.9)	1.6(0.8-3.2)		
65 years and over	11/27(40.7)	1.5(0.6-4)		
**Hospital of diagnosis**				
Other hospitals	61/156(39.1)	1	1	
Hospital A	62/165(37.6)	0.9(0.6-1.4)	1.0(0.6-1.7)	0.992
Hospital B	130/244(53.3)	1.7(1.1-2.7)	2.2(1.3-3.6)	0.002
Hospital C	25/97(25.8)	0.5(0.3-0.9)	1.0(0.6-2.1)	0.759
Hospital D	27/69(39.1)	1(0.8-1.8)	2.4(1.2-4.9)	0.012
Hospital E	81/229(35.4)	0.8(0.6-1.3)	0.9(05-1.5)	0.755
**Geographical area of origin**				
Latin America	80/356(22.5)	1	1	
India-Pakistan	134/249(53.8)	4.0(2.8-5.7)	2.0(1.3-3.2)	0.002
North Africa	67/131(51.1)	3.6(2.4-5.5)	2.0(1.2-3.3)	0.005
Other countries	105/224(46.9)	3.0(2.1-4.4)	1.8(1.2-2.8)	0.006
**IDU^e^**				
No	355/918(38.7)	1		
Yes	31/42(73.8)	4.5(2.2-9)		
**District of residence**				
Other districts	171/552(31)	1	1	
Inner-city	173/358(48.3)	2.0(1.5-2.7)	1.3(0.9-1.8)	0.203
Unknown	42/50(84)	11(5.4-25.4)	4.4(1.8-10.7)	0.001
**HIV**				
No	331/875(37.8)	1		
Yes	55/85(64.7)	3.0(1.9-4.8)		
**Smoking**				
No	251/661(38)	1		
Yes	135/299(45.2)	1.3(1.0-1.8)		
**Use of alcohol**				
No	321/825(38.9)	1		
Yes	65/135(48)	1.5(1.0-2.1)		
**Incarceration history**				
No	360/927(38.8)	1	1	
Yes	26/33(78.8)	5.9(2.5-13.6)	3.8(1.4-10.4)	0.008
**Homeless**				
No	326/883(36.9)	1	1	
Yes	60/77(77.9)	6.0(3.5-10.5)	5.6(3.0-10.6)	< 0.001
**Type of TB^f ^of index case**				
Pulmonary smear-positive	97/353(27.5)	1	1	
Pulmonary smear (-) culture (+)	68/199(34.2)	1.3(0.9-2.0)	1.3(0.8-2.0)	0.253
Pulmonary culture (-)	60/144(41.7)	1.9(1.3-2.8)	2.1(1.2-3.5)	0.005
Extrapulmonary	160/263(60.8)	4.1(3.0-5.8)	3.0(1.8-5.0)	< 0.001
**Chest X-ray**				
Cavitary	64/239(26.8)	1	1	
Normal	97/155(62.6)	4.6(3.0-7.0)	2.0(1.1-3.9)	0.021
Non-cavitary	217/555(39.1)	1.8(1.3-2.5)	1.2(0.8-1.8)	0.399
Not performed	8/11(72.7)	7.2(1.9-28.3)	3.0(0.7-13.9)	0.157

Barcelona 2000-2005

^a^Contact tracing, ^b^Odds ratio, ^c ^Adjusted odds ratio, ^d^After the introduction of community health workers intervention, ^e^injecting drug user, ^f^Tuberculosis

## Discussion

There was a low contact tracing coverage within the immigrant population during the pre-intervention period. The main reason for that was that the TBPCP was not prepared to manage the large influx of immigrants that ocurred during this period. Moreover, a considerable percentage of the immigrants came from high TB endemic countries and did not speak Spanish. This study shows that immigration is a dynamic phenomenon. In the second period there were fewer patients from North Africa and more young adults. We have also found a statistically significant increase in performed contact tracing among immigrants after the incorporation of CHW. This increase suggests that CHW contributed considerably to the improvement of the prevention activities, due to their communication with cases and their contacts by interpreting and mediating for clinical care and in the community [17,18].

Regarding factors associated with lack of contact tracing, the two specific hospitals which were identified deal with large numbers of immigrants, did not have the appropriate means to perform contact tracing and frequently refered patients to a family doctor for contact tracing. Countries of origin such as India, Pakistan, Maghreb and other non-Latin American countries were also associated to lack of contact tracing performed, possibly because the language skills and the cultural barriers that may influence patient's behaviour in relation to TB. Other factors were homeless and unknown residence. CHW contacted some cases, such as the homeless and those with no known residence, by phone or in person. A lack of contact tracing for all forms of TB was associated with male sex, history of imprisonment, extrapulmonary TB and a normal CXR. Among the few number of patients with incarceration history, the percentage of those without contact tracing reach 78.8%, some of these patients were HIV-infected IDU. The risk factors found in our study are similar to those reported in other studies [19,20]. It is important to note that the lack of intervention of CHW is associated with lack of contact tracing in all TB cases and in the sub-group of smear positive cases.

International recommendations from organisations such as the WHO, the Centers for Disease Control and Prevention and the International Union Against Tuberculosis and Lung Disease, suggest the incorporation of health providers, community health promoters, social health workers and outreach health workers in areas with high levels of immigration or with many ethnic groups [21-23]. The coordinated action of CHW with PHN and TBPCP doctors has contributed to locating cases and their contacts, as well as to increase treatment adherence. They have improved access to healthcare by ensuring that each patient and their contacts can obtain an individual health insurance card. In our study, the influence of CHW on TB treatment adherence was limited because the percentage of treatment adherence in Barcelona was already satisfactory due directly observed therapy in higher risk patients since 1995 [24].

Mass migration has affected the epidemiology of TB. In Spain, a consensus document has been developed to address this problem, even in those with no right of residence. This policy recommends that all migrants have a health card, an initial medical examination at their first appointment and to include CHW in TB control programmes [12,25].

From a multidisciplinary perspective, the incorporation of CHW can reinforce the effectiveness of PHN personnel and minimise difficulties accessing care [26]. Similarly, the "IEC" approach (information, education, communication) develops both care and community level actions, such as health promotion in TB [27,28]. The educational sessions in private homes and associations for immigrants have reached the target population in their daily settings outside of working hours [29]. Mediation, conflict resolution, linguistic translation and cultural interpretation has improved the relationship between patients and health care personnel and has reduced communication related issues.

The CHW strategy ensures that patients who were from a different culture are supported, accompanied and defended confronted with TB stigma and social and occupational discrimination. The strategy offers community-based educational support in which patients are actors controlling TB transmission and confirms that TB is, above all, a social process involving multiple context-related factors of healing and control over transmission [30,31].

One study limitation was the variation in characteristics between both periods; an increase of cases between 25-39 years of age, from Latin America and India, Pakistan and from inner-city in the CHW group. The increase in immigrants would most likely have worsened contact tracing and therefore our figures may have underestimated the benefit of the CHW intervention. Eight percent of cases were not contacted, despite multiple phone calls and home visits. However, given the high mobility of immigrant groups, this is considered a low percentage.

The effectiveness of TB programs depends upon their ability to adapt to the emerging needs of the population changes. Therefore, it is recommended incorporate CHW into every TB program with the goal of improving TB control in immigrant populations. This can also be extended to other infectious diseases such as HIV, sexually transmitted diseases and malaria. CHW incorporation can also save social and economic costs in TB programs, however studies on cost-effectiveness of the CHW interventions in the TB programs are also necessary [32].

## Conclusions

We conclude that TB programs in areas of high immigration can improve their effectiveness by the incorporation CHW who act in coordination with the PHN and other professionals. They would act as interpreters and inter-cultural mediators as well as undertake community actions which positively reinforce the response from patients, as seen in the improvements in contact tracing. This is possible when immigrant people have confidence, both linguistically and culturally, is the response of any human being when you feel welcomed and accompanied. The findings of this study encourage us to strengthen the interdisciplinary work by CHW.

## Ethics approval statement

Demographic and clinical data were obtained from the epidemiological questionnaire used by TB Prevention and Control Program (TBPCP). TB is a mandatory notification disease and community health workers and public health nurses need to know the name of the patients in order to make the interviews. The data was treated and analysed anonymously. The analysis was carried out retrospectively and involved data collected on a routine basis within the National Tuberculosis Program approved by the Spanish Ministry of Health. Therefore, no ethical approval nor informed consent was required. All data were treated in a strictly confidential manner following the ethical principles of the Helsinki Declaration of 1964 revised by the World Medical Organization in Edinburgh, 2000 and the Organic Law 15/1999 of Data Protection in Spain. Declaración de Hèlsinki de la Asociación Médica Mundial: http://www.isciii.es/htdocs/terapia/documentos/Declaracion_de_Helsinki.pdf. Ley Orgánica española de protección de datos 15/1999: http://noticias.juridicas.com/base_datos/Admin/lo15-1999.html.

## Competing interests

The authors declare that they have no competing interests.

## Authors' contributions

OJE participated as principal investigator; OA participated in the design and conception of the study, statistical analysis and coordination; MJP conducted the fieldwork and helped to draft the manuscript; SF participated in the design, conception of the study and the final draft of the manuscript; CM participated in the design of the study and performed the statistical analysis and CJ participated in the design and conception of the study and overall coordination of the same. All authors read and approved the final manuscript.

## Pre-publication history

The pre-publication history for this paper can be accessed here:

http://www.biomedcentral.com/1471-2458/12/158/prepub
